# Association between FOXP3 Expression and Disease-Free Survival in Triple Negative Breast Cancer

**DOI:** 10.30699/ijp.2025.2052684.3418

**Published:** 2025-08-15

**Authors:** Himawan Adhitama, Suwardjo Suwardjo, Ery Kus Dwianingsih, Teguh Aryandono

**Affiliations:** 1 Department of Surgery, Surgical Oncology Division, Faculty of Medicine, Public Health, and Nursing, Gadjah Mada University/Dr. Sardjito Hospital, Yogyakarta, Indonesia; 2 Department of Anatomical Pathology, Faculty of Medicine, Public Health, and Nursing, Gadjah Mada University/Dr. Sardjito Hospital, Yogyakarta, Indonesia

**Keywords:** FOXP3 Protein, Disease-Free Survival, Triple Negative Breast Neoplasms

## Abstract

**Background & Objective:**

Triple-negative breast cancer (TNBC) is an aggressive subtype lacking estrogen, progesterone, and HER2 receptors, often leading to poor prognosis due to high recurrence and metastasis. FOXP3 expression, associated with suppressed anti-tumor immunity, is a potential prognostic marker in TNBC. However, the association between FOXP3 expression and disease-free survival (DFS) in TNBC remains controversial. This study aims to explore the relationship between FOXP3 expression and DFS in TNBC patients, contributing to the understanding of its prognostic significance.

**Methods:**

This retrospective study included 47 TNBC patients. Immunohistochemistry examination assessed FOXP3 expression, which was then categorized into low and high expression based on an optimal cut-off point. Univariate and multivariate analyses were conducted using Cox regression test to determine association between variables and DFS. Survival analysis was assessed using the Kaplan-Meier method and the log-rank test.

**Results:**

All patients were female mostly over 50 years old (66%). The majority had tumors >2 cm (87.2%) and LVI (66%). High-grade tumors (grade 3) were predominant (93.6%). Most patients showed moderate TIL levels (91.5%). No significant associations were found between FOXP3 expression and age (p=0.253), tumor size (p=0.308), lymph node status (p=0.466), tumor grade (p=0.476), LVI (p=0.422), patient's stage (p=0.644), and TILs (p=0.783). However, high FOXP3 expression was significantly associated with worse DFS (p=0.019). Additionally, a higher stage was associated with worse DFS (p=0.002).

**Conclusion:**

High FOXP3 expression is associated with lower DFS in TNBC patients. FOXP3 expression was not associated with age, tumor size, lymph node status, tumor grade, LVI, TILs, or cancer stage.

## Introduction

Breast cancer is the most frequently diagnosed cancer in women and ranks as the second leading cause of cancer-related deaths worldwide, following lung cancer (1). Mortality rates are exceptionally high in low- and middle-income countries, including Indonesia, primarily due to late diagnoses, often when the disease has already metastasized (2,3).

Breast cancer is classified based on the expression of the estrogen receptor (ER), progesterone receptor (PR), and HER2. Triple-negative breast Cancer (TNBC), a subtype of breast cancer, is characterized by the absence of these three markers. TNBC is more common in women under 40 years old and has a recurrence rate of 25% after surgery. It is also a highly invasive form of breast cancer, with 46% of cases involving distant metastases, often in the brain and visceral organs. The average survival period for metastatic patients is only 13.3 months (4). 

The poor prognosis of TNBC is largely attributed to its immunogenic nature, which results from its unstable genomic makeup and numerous mutations. Additionally, TNBC exhibits higher levels of stromal tumor-infiltrating lymphocytes (TILs) and elevated expression of programmed death ligand 1 (PD-L1) (5).

TILs play a critical role in the tumor microenvironment as effectors of adaptive host immune responses, influencing cancer growth and progression. Consequently, TILs serve as predictive and prognostic biomarkers in TNBC. Several studies have shown that higher TIL levels are associated with higher pathological complete response (pCR) rates in patients receiving neoadjuvant chemotherapy and correlate with improved overall survival in TNBC (5,6).

Previous studies have suggested that different components of TILs are associated with varying prognoses. One of the components of TILs, T regulators (Tregs), is a heterogeneous group of T cells with distinct phenotypes and functions, playing a crucial role in suppressing antitumor immune responses. Tregs modulate the immune system, maintain tolerance to self-antigens, and prevent autoimmune diseases. Tregs are characterized by the expression of the interleukin-2 receptor α chain (CD25) and Forkhead box protein 3 (FOXP3), with FOXP3 being a key regulator of Treg development and function, making it the most specific Treg marker (5). Studies analyzing FOXP3 expression in breast cancer using immunohistochemistry have demonstrated reliable and accurate localization and visualization of FOXP3 protein within tissue sections, allowing the identification of FOXP3 expression in specific tumor regions or in tumor-infiltrating immune cells such as regulatory T cells (Tregs) (7,8). High FOXP3 expression in breast cancer patients is often linked to a poor prognosis (9,10,11).

A previous study found that patients with tumors expressing high levels of FOXP3 Tregs had a lower 5-year disease-free survival (DFS) rate compared to those with low FOXP3 Treg expression (85.7% vs. 98.5%, respectively) (12). However, the role of FOXP3 in determining prognosis remains controversial. Some studies suggest that FOXP3 presence makes tumors less responsive to cytotoxic chemotherapy, as seen in ER+ breast cancer, while other studies indicate that higher FOXP3 expression is associated with better outcomes, particularly in HER2+/ER subtype breast cancer (5,9). This study aims to clarify the significance of FOXP3 in breast cancer prognosis by analyzing the association between FOXP3 expression and clinicopathological status, TILs, and disease-free survival (DFS).

## Materials and Methods

### Study Population

This study employed a retrospective cohort design, involving breast cancer patients diagnosed with triple-negative breast cancer (TNBC) at Surgical Oncology Outpatient in Dr. Sardjito Hospital from July 2016 to July 2020. Samples meeting inclusion and exclusion criteria were analyzed retrospectively using medical record data. Secondary data collected included clinicopathological characteristics, tumor-infiltrating lymphocytes (TIL), FOXP3 expression in histopathological examinations, cancer stage, and findings from radiological investigations (breast ultrasound, chest X-ray, and abdominal ultrasound). Disease-free survival (DFS) was assessed during follow-up visits, with recurrence or distant metastases confirmed through pathological or radiological evaluations. Patients with stage IV disease, samples with minimal tumor content and extensive necrosis, and unusable or damaged tissue specimens were excluded. 

### Immunohistochemistry Examination

According to established inclusion and exclusion criteria, the study utilized Formalin Fixation and Paraffin Embedding (FFPE) samples obtained from the study population. Each sample was sectioned into 3-micron thick slices, mounted on poly-L-lysine-coated slides. Slides were incubated at 37°C overnight and heated at 60°C for 10 minutes and subjected to immunohistochemical analysis. Deparaffinization was performed using xylene (three changes, 5 minutes each), followed by rehydration through graded alcohols (96%, 70%, 50%) and rinsing in running water. Antigen retrieval was carried out in Tris-EDTA buffer (pH 9.0) at 95°C for 20 minutes using a pressure cooker, followed by cooling at room temperature. Slides were washed in PBS (pH 7.4) and incubated with peroxidase block, super block, and then with anti-FOXP3 primary antibody. Subsequently, UltraTek Anti-Polyvalent and UltraTek HRP were applied. Detection was performed using DAB (3,3′-diaminobenzidine) and counterstained with hematoxylin. Slides were then treated with bluing reagent, dehydrated through graded alcohols, cleared with xylene, and mounted with Ez Mount and cover glass. Immunohistochemical staining results were evaluated by two independent observers using an Olympus CX23 microscope. Normal human tonsil cells were the positive control for FOXP3 staining, while the negative control was established by omitting the primary antibody. FOXP3 expression was quantified across ten visual fields using ImageJ software. 

### Statistical analysis

The optimal cut-off value for determining high and low FOXP3 expression was established using the Maximally Selected Rank Statistics method within the Maxstat program (Jamovi statistical software version 2.3.26) (13,14,15). The Chi-square test assessed the relationship between clinicopathological profiles and the independent variables. Survival analysis was conducted using the Kaplan-Meier method and the log-rank test. All statistical analyses were performed using IBM SPSS Statistics version 26. A p-value of <0.05 was considered statistically significant.

## Results

### Patient Characteristics

This study included 47 female breast cancer patients with the triple-negative breast cancer (TNBC) subtype, as described in Table 1. All subjects were female, with the majority being over 50 years old (31 patients, 66%). Most tumors were larger than 2 cm in 41 patients (87.2%). Lymphovascular invasion (LVI) was identified in 31 patients (66%). The most prevalent tumor grade was grade 3, found in 44 patients (93.6%), and followed by grade 2 in 3 patients (6.4%), with no patients diagnosed with stage 1 cancer. Tumor-infiltrating lymphocytes (TILs) were low in 4 patients (8.5%), while 43 patients (91.5%) had moderate TILs. There were no cases with high TIL levels.

**Table 1:** Patients characteristics

No	Characteristic		Number of cases	Percentage
1	Age	≤	16	34%
		>50 years old	31	66%
2	Tumor Size	≤	6	12.8%
		>2 cm	41	87.2%
3	Lymph node status	Positive	31	66%
		Negative	16	34%
4	Grade	Grade 1	0	0
		Grade 2	3	6.4%
		Grade 3	44	93.6%
5	Lymphovascular Invasion (LVI)	Positive	31	66%
		Negative	16	34%
6	Stage	Stage 1	0	0
		Stage 2	14	29.8%
		Stage 3	33	70.2%
7	Tumor Infiltrating Lymphocytes (TILs)	Low	4	8.5%
		Medium	43	91.5%
		High	0	0

### FOXP3 Expression Analysis

**Fig 1:**
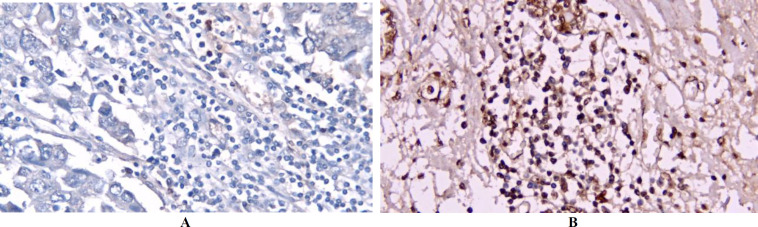
IHC analysis of FOXP3 expression in FFPE samples of TNBC. (a) representative image of tumors with low FOXP3 expression, (b) representative image of tumors with high FOXP3 expression

FOXP3 expression, primarily nuclear, was evaluated using immunohistochemistry (IHC) in 47 FFPE samples from TNBC patients (Figure 1). The analysis determined an optimal cut-off value of 2.13% for FOXP3 expression. Samples with ≤2.13% TILs were categorized as low expression, and those with >2.13% as high expression. Low FOXP3 expression was observed in 15 patients (31.9%), while high expression was found in 32 patients (68.1%). No significant associations were found between FOXP3 expression and age (p=0.465), tumor size (p=0.936), lymph node status (p=0.555), tumor grade (p=0.182), LVI (p=0.211), cancer stage (p=0.716), or TILs (p=0.417) (Table 2).

**Table 2:** Association Between Clinicopathological Characteristics and FOXP3 Expression

No	Characteristic		FOXP3 Expression		*p-value*
			Low	High	
1	Age	50 years old	4	12	0.465
		>50 years old	11	20	
2	Tumor Size	2 cm	2	4	0.936
		>2 cm	13	28	
3	Lymph node status	Positive	9	22	0.555
		Negative	6	10	
4	Grade	Grade 2	2	1	0.182
		Grade 3	13	31	
5	Lymphovascular Invasion (LVI)	Positive	8	23	0.211
		Negative	7	9	
6	Stage	Stage 2	5	9	0.716
		Stage 3	10	23	
7	Tumor Infiltrating Lymphocytes (TILs)	Low	2	2	0.417
		Medium	13	30	

### Survival Analysis

**Fig 2:**
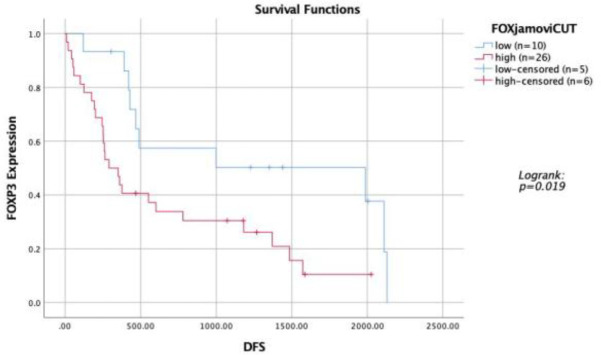
Kaplan Meier Curve showing survival analysis of TNBC patients with high vs low tumoral FOXP3 expression

**Fig 3:**
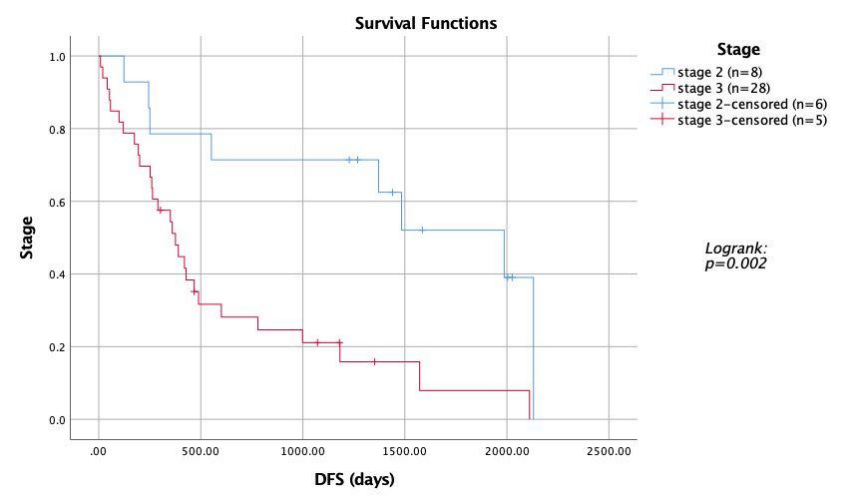
Kaplan Meier Curve showing survival analysis of TNBC patients with different stages

Survival analysis revealed that patients with low FOXP3 expression had better disease-free survival (DFS), with a median DFS of 1,987 days compared to 290 days in patients with high FOXP3 expression (p=0.019) (Figure 2). Additionally, patients diagnosed at stage 2 had better DFS than those at stage 3 (1,987 days vs. 376 days, p=0.002) (Figure 3). Univariate analysis indicated that both the stage at diagnosis and FOXP3 expression significantly influenced DFS (Table 3). Patients at stage 2 had a significantly better DFS than those at stage 3 (p=0.003). High FOXP3 expression was statistically significant and associated with worse DFS (p=0.023). Other factors, including age, tumor size, lymph node status, grade, LVI, and TILs, did not show statistically significant results.

### Multivariate Analysis

Multivariate analysis confirmed that patients at stage 2 had significantly better DFS than those at stage 3 (p=0.015). High FOXP3 expression was again associated with worse DFS (p=0.013). Other clinicopathological parameters, such as age, tumor size, lymph node status, grade, LVI, and TILs, did not yield statistically significant findings (Table 3).

**Table 3:** Univariate and Multivariate Analysis

No	Characteristics	Univariate HR (95% CI)	*p-value*	Multivariate HR (95% CI)	*p-value*
1	Age				
	50 years old	1,231 (0,618-2,454)	0.555	0,947 (0,410-2,185)	0.898
	>50 years old	1 (*Reference*)		1 (*Reference*)	
2	Tumor Size				
	2 cm	1 (*Reference*)	0.724	1 (*Reference*)	0.687
	>2 cm	1,209 (0,422-3,462)		0,765 (0,207-2,819)	
3	Lymph node status				
	Positive	1,882 (0,891-3,977)	0.097	0,889 (0,312-2,535)	0.825
	Negative	1 (*Reference*)		1 (*Reference*)	
4	Grade				
	Grade 2	1 (*Reference*)	0.395	1 (*Reference*)	0.657
	Grade 3	1,866 (0,443-7,850)		0,677 (0,121-3,789)	
5	Lymphovascular Invasion (LVI)				
	Positive	0,785 (0,401-1,538)	0.481	0,643 (0,293-1,412)	0.272
	Negative	1 (*Reference*)		1 (*Reference*)	
6	Stage				
	Stage 2	1 (*Reference*)	0.003	1 (*Reference*)	0.015
	Stage 3	3,660 (1,538-8,709)		4,309 (1,333-13,923)	
7	Tumor Infiltrating Lymphocytes (TILs)				
	Low	1,186 (0,358-3,933)	0.780	0,945 (0,219-4,070)	0.939
	Medium	1 (*Reference*)		1 (*Reference*)	
8	FOXP3				
	Low	1 (*Reference*)	0.023	1 (*Reference*)	0.013
	High	2,544 (1,136-5,698)		3,085 (1,272-7,481)	

## Discussion

Our analysis revealed no significant association between FOXP3 expression and clinicopathological features such as age, tumor size, lymph node status, tumor grade, LVI, TILs, or tumor stage. While no studies have specifically linked age with FOXP3 expression in the TNBC subtype, a survey by Berben et al. (2020) in luminal B breast cancer suggested that older patients tend to have a higher density of FOXP3 infiltration in their tumors (16). Our findings contrast with those of Takenaka et al. (2013), who found a significant association between FOXP3 expression and larger tumor size (17). This discrepancy might be due to the sample size and staging differences between the studies; Takenaka et al.'s study included 100 patients, predominantly in stages 1 and 2, whereas our study included 47 patients, most of who were in stage 3, with 87.2% presenting with larger tumors. The relatively low number of 47 cases in our study reflects the rarity of triple-negative breast cancer (TNBC) over the four-year study period. TNBC represents a minority of all breast cancer cases, comprises approximately 10-15% of all breast cancer diagnoses. As our institution serves as a tertiary referral center, the patient population is diverse, but TNBC remains relatively uncommon.

Bates et al. (2006) reported a positive association between FOXP3 expression, high tumor grade, and positive lymph node status (18). However, their study involved 287 patients and included various breast cancer subtypes, which may account for the difference in findings. Additionally, Glajcar et al. (2021) noted that tumors with LVI had significantly higher Treg infiltration, but their study focused on luminal A breast cancer, whereas ours was centered on TNBC (19). Furthermore, Indiralia et al. (2018) observed that FOXP3 expression was higher at early stages (T1 and T2) and decreased at advanced stages (T3 and T4) (20), while Sudarsa et al. (2019) reported that high FOXP3 expression was not associated with advanced stage TNBC. Still, low CD8 expression significantly increased the risk of advancing to a higher stage (21).

We found no significant relationship between the number of TILs and FOXP3 expression. FOXP3 is a specific marker for Tregs, a subset of TILs, but high FOXP3 expression does not directly correlate with the overall TIL count, as TILs comprise various types of T cells, including CD8-positive cells. The prognostic significance of FOXP3 remains debated, with some studies linking it to poorer outcomes due to its role in suppressing antitumor immunity. In contrast, others, such as DeLeeuw et al. (2012), have demonstrated positive prognostic value in specific contexts. The variability in FOXP3's prognostic significance may be influenced by factors such as tumor location, molecular subtype, cancer stage, and the tumor microenvironment (22,23).

Our survival analysis showed that patients with low FOXP3 expression had significantly better disease-free survival (DFS), with a median DFS of 1,987 days compared to 290 days for patients with high FOXP3 expression. These findings align with a meta-analysis by Shou et al. (2016), which concluded that high FOXP3 expression is associated with poorer survival in hormone-positive breast cancer. However, this contrasts with the findings of Jiang et al. (2015) and Yeong et al. (2017), who reported that high FOXP3 expression in TNBC is associated with better survival (24,25).

Univariate analysis identified the patient’s stage at diagnosis (p=0.003) and FOXP3 expression (p=0.023) as statistically significant factors. Multivariate analysis further confirmed these findings, with stage (p=0.015) and FOXP3 expression (p=0.013) being independent prognostic factors. These results are consistent with studies by Balsari et al. (2009), which identified FOXP3 expression as an independent prognostic factor in breast cancer (26), and by Aryandono (2006), which highlighted the significant impact of cancer stage on patient survival (27).

This study has several limitations. It is a retrospective cohort study, and the findings need to be validated in prospective studies. Additionally, there needs to be a standardized method for assessing FOXP3 expression, leading to discrepancies with other studies. For example, Mahmoud et al. (2011) and Merlo et al. (2009) categorized FOXP3 expression as positive or negative (26,28), while Takenaka et al. (2013) used a percentage-based scoring system (17), and Jamiyan et al. (2020) classified FOXP3 expression as high or low based on the median cut-off of positive cell numbers (6). 

Moreover, this study only focused on FOXP3, a subset of TILs. Future research should evaluate and compare other TIL subsets, such as CD4, CD8, and PDL1, to better understand their roles in TNBC.

## Conclusion

In conclusion, high FOXP3 expression was significantly associated with lower disease-free survival in TNBC patients. However, in these patients, FOXP3 expression was not associated with age, tumor size, lymph node status, tumor grade, LVI, TILs, or cancer stage.

## Data Availability

There is no additional data separate from available in cited references.
